# Real-time endoscopic image orientation correction system using an accelerometer and gyrosensor

**DOI:** 10.1371/journal.pone.0186691

**Published:** 2017-11-03

**Authors:** Hyung-Chul Lee, Chul-Woo Jung, Hee Chan Kim

**Affiliations:** 1 Department of Anesthesiology and Pain Medicine, Seoul National University Hospital, Seoul, Korea; 2 Department of Biomedical Engineering, College of Medicine, Seoul National University, Seoul, Korea; 3 Institute of Medical and Biological Engineering, Medical Research Center, Seoul National University, Seoul, Korea; University Hospital Llandough, UNITED KINGDOM

## Abstract

The discrepancy between spatial orientations of an endoscopic image and a physician’s working environment can make it difficult to interpret endoscopic images. In this study, we developed and evaluated a device that corrects the endoscopic image orientation using an accelerometer and gyrosensor. The acceleration of gravity and angular velocity were retrieved from the accelerometer and gyrosensor attached to the handle of the endoscope. The rotational angle of the endoscope handle was calculated using a Kalman filter with transmission delay compensation. Technical evaluation of the orientation correction system was performed using a camera by comparing the optical rotational angle from the captured image with the rotational angle calculated from the sensor outputs. For the clinical utility test, fifteen anesthesiology residents performed a video endoscopic examination of an airway model with and without using the orientation correction system. The participants reported numbers written on papers placed at the left main, right main, and right upper bronchi of the airway model. The correctness and the total time it took participants to report the numbers were recorded. During the technical evaluation, errors in the calculated rotational angle were less than 5 degrees. In the clinical utility test, there was a significant time reduction when using the orientation correction system compared with not using the system (median, 52 vs. 76 seconds; *P* = .012). In this study, we developed a real-time endoscopic image orientation correction system, which significantly improved physician performance during a video endoscopic exam.

## Introduction

Video endoscopes, such as laparoscopes, gastroenteroscopes, and bronchoscopes, are used in many fields of medicine to help clinicians diagnose and treat patients less invasively with shorter hospital stays. However, video endoscopes are difficult to operate, and it takes time for clinicians to learn how to interpret endoscopic images. One of the difficulties is the discrepancy between the spatial orientation of the endoscopic images and the endoscopist’s working environment [1, 2]. For example, it has been reported that a surgeon’s performance decreases when the optical axis of the endoscope equipment does not match the direction of gravity [3, 4]. Attempts to solve this problem have included pattern recognition [5], electromagnetic field sensors [6], and accelerometers [7, 8]. With the pattern recognition method, various computer algorithms were used to extract the features from fiducial markers or anatomical structures on the endoscopic image [5, 9]; however, this method cannot be used when there is no target in the line of sight. The electromagnetic method uses a current induced by a coil moving through a magnetic field. This technique is widely used as a tracking system for image-guided interventions [10], but it can also be used for image orientation correction [6]. However, conductive surgical instruments or electromagnetic interference, from x-ray fluoroscopy for example, can distort the magnetic field and influence the measurements [11]. The accelerometer method uses a low-cost, small inertial sensor to correct the direction of endoscopic images [7, 8]; however, it cannot distinguish between translational acceleration and gravity. Therefore, an accelerometer should be used in combination with a gyrosensor to correct the direction of a rapidly rotating endoscope.

The gyrosensor has a fast response time that is not influenced by translational acceleration. Because the accelerometer fails to differentiate acceleration from gravity, the gyrosensor measures the rotational angle more accurately than the accelerometer when the endoscope rotates. Moreover, the integration error of the rotational angle from the gyrosensor that accumulates over time can be easily corrected using an accelerometer signal. Therefore, these two sensors are often used to complement each other.

In this study, we developed and evaluated a real-time system for correcting endoscopic image orientation using an accelerometer and gyrosensor.

## Materials and methods

### Development of the system

To obtain the direction of gravity and angular velocity, an accelerometer and gyrosensor were attached to the handle of the endoscope. We assumed that the endoscope handle was a rigid body and that there would be no twisting from the handle to the tip of the endoscope. Quaternion, direction cosine matrix, Euler angles, and axis-angle representation can be used to signify the posture of a rigid body. Due to its simplicity and clear physical meaning, we used the axis-angle to represent the posture of the endoscope handle. The axis was defined as a unit vector indicating the direction of the advancing endoscope. The rotational angle θ was defined as 0 when the sensor was in the opposite direction of gravity and increased when the endoscope rotated in the clockwise direction. Because a singularity occurs when the operator holds the endoscope handle perpendicular to the ground, this action was prohibited during the study.

### Calculation of the rotational angle

**Fig 1:**
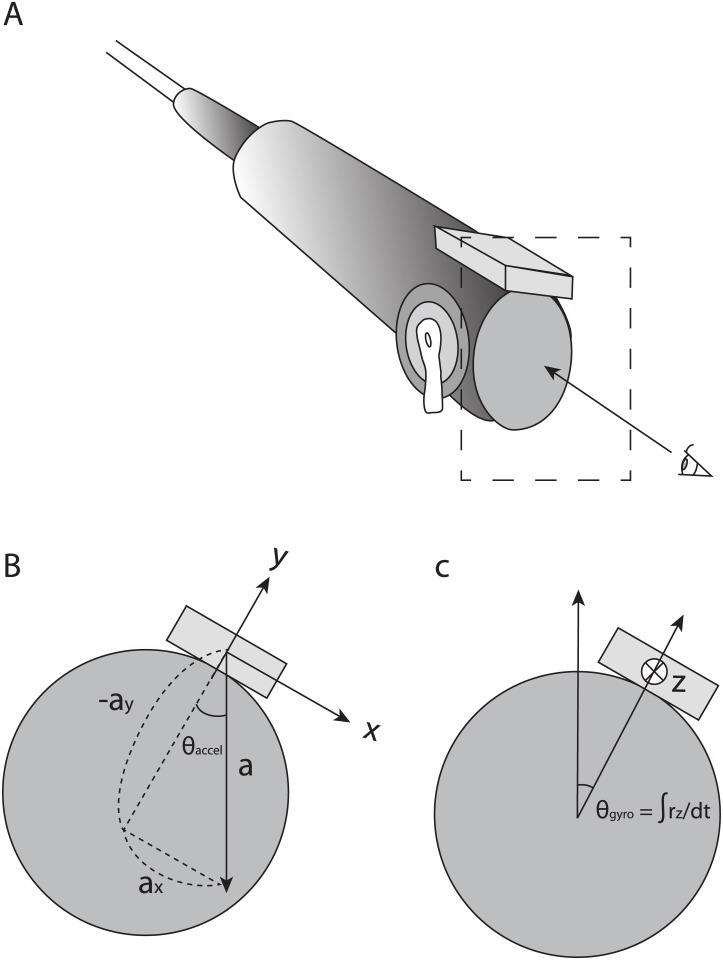
Axes definitions. A. the sensor attachment and the viewpoint of the observer B. axes for the accelerometer. a_x,y_ is the acceleration measured by the accelerometer and θ_accel_ is the rotational angle calculated from the accelerometer data. C. axes for the gyrosensor. r_z_ is the angular velocity measured by the gyrosensor and θ_gyro_ is the rotational angle calculated from the gyrosensor data.

The sensor was attached so that the *x* axis pointed to the right as seen by the operator, the *y* axis pointed toward the head of the endoscope, and the *z* axis point in the direction of the advancing endoscope (Fig 1).

The rotational angle with respect to the direction of gravity was calculated as:

$$\theta_{accel}=atan2(a_{x},-a_{y})$$ (1)

$$\theta_{zyro}=\int r_{z}$$ (2)

where *θ*_accel_ and *θ*_zyro_ are the rotational angles from the accelerometer and gyrosensor, respectively; *a*_*x*,*y*_ is the acceleration from the accelerometer, *r*_*z*_ is the angular velocity from the gyrosensor, and atan2 (*y*,*x*) is a function that returns the arc tangent of *y*/*x* within the range of −*π* and *π* depending upon the sign of *x* and *y*.

The rotational angles from the accelerometer and gyrosensor were calibrated using a Kalman filter [12, 13]. The filter used the output of the gyrosensor and Eq (3).

$$Model\;equation:\;\theta_{Kalman,k}=\theta_{Kalman,k-1}+(T\times r_{k})+w_{k}$$ (3)

where *θ*_Kalman_ is a state variable–the rotational angle after the Kalman filter (*θ*_Kalman,0_ = *θ*_accel,0_), *r*_k_ is the angular velocity from the gyrosensor, *T* is the time from the last measurement, *w*_k_ is the processing error, and *θ*_accel_ is the rotational angle from the accelerometer.

Eq (4) represents the measurement equation of the Kalman filter using the accelerometer output.

$$Measurement\;equation:\theta_{accel,k}=\theta_{Kalman,k}+z_{k}$$ (4)

where *θ*_accel_ is a measurement variable–the rotational angle from the accelerometer, *θ*_Kalman,k_ is a state variable, and *z*_k_ is the measurement error.

In the Kalman filter algorithm, w_k_ and z_k_ were assumed to be zero mean Gaussian white noise with a covariance of Q_k_ and R_k_. The smaller the Q_k_ value, the greater the importance of the gyrosensor output; the smaller the R_k_ value, the greater the importance of the accelerometer output. We set the R_k_ value to give less weight to the accelerometer output if an additional acceleration was observed.

$$Q_{k}=0.1$$ (5)

$$R_{k}=500,\;when|a_{k}|<1.0001g$$

$$R_{k}=50,000,\;when\;1.0001g<|a_{k}|<1.02g$$

$$R_{k}=1,000,000,\;when\;1.02g<|a_{k}|$$

where *a*_k_ is the acceleration from the accelerometer and *g* is the acceleration of gravity.

The predicted rotational angle was calculated using Eq (6)

$${\hat{\theta }}_{\;Kalman,k}={\hat{\theta }}_{\;Kalman,k-1}+(T\times r_{k})$$ (6)

$$P_{k}=P_{k-1}+Q_{k}$$

where $\hat{\theta }$_Kalman,k_ is the estimated rotational angle from the Kalman filter, r_k_ is the angular velocity from the gyrosensor, and P_k_ is the estimated covariance (P_0_ = 1).

Finally, the states and the estimated covariance were updated for the next iteration as shown in Eq (7).

$$K_{k}=P_{k}/(P_{k}+R_{k})$$ (7)

$${\hat{\theta }\;}_{Kalman,k}=(1-K_{k})\times {\hat{\theta }\;}_{Kalman,k}+K_{k}\times \theta_{accel,k}$$

$$P_{k}=(1-K_{k})\times P_{k}$$

where K_k_ is the Kalman gain.

**Fig 2:**
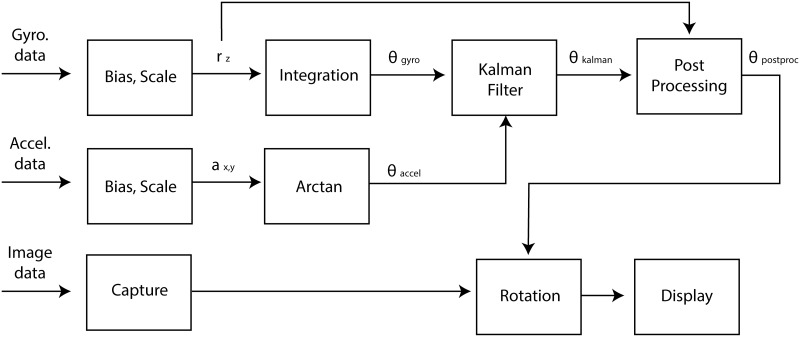
Schematic diagram of the image orientation correction system. *r*_*z*_ is the angular velocity measured by the gyrosensor; *a*_*x*,*y*_ is the acceleration measured by the accelerometer; *θ*_gyro_ and *θ*_accel_ are the rotational angles calculated from the gyrosensor and accelerometer data, respectively; *θ*_Kalman_ is the rotational angle after the Kalman filter; and *θ*_postproc_ is the final rotational angle used for image rotation. Gyro., gyrosensor; Accel., accelerometer.

Subsequently, the rotational angle from the Kalman filter was postprocessed to correct for the delay between the image acquisition and sensor measurement (Fig 2). Because the imaging device and the sensor have different processing and data transfer times, when the image arrives at the processor, the last measurement data from the sensor should not be used without correction. If we assume that the time difference between the last available sensor output and image frame does not change during the exam, the correction should be as shown in Eq (8):

$$\theta_{postproc,k}=\theta_{Kalman,k}-(T_{d}\times r_{k})$$ (8)

where *θ*_postproc,k_ is the final rotational angle after postprocessing, r_k_ is the angular velocity from the gyrosensor, and *T*_d_ is the time difference between the last available sensor output and image frame. *T*_d_ was determined by a least square error method using the pre-examination data from the system with Eq 9.

$$T_{d}={\left(r^{T}r\right)}^{-1}r^{T}(\theta {}_{Kalman}-\theta_{optical})$$ (9)

where **r** is the vector of the measured angular velocity, ***θ***_**Kalman**_ is the rotational angle vector from the Kalman filter, and ***θ***_**optical**_ is the optical rotational angle vector (method will be described in the Technical evaluation section).

### Devices used for the system

A 3-Space Sensor™ Bluetooth (Yost Engineering Inc, Portsmouth, OH, USA) was used for the system. According to the manufacturer’s specifications, the accelerometer and gyrosensor precisions are 0.0024 m/s^2^ and 0.070°/s, respectively. The sensor device also has a magnetometer, although it was not used in this study. The video was captured by a charge-coupled device (CCD) camera attached to the eyepiece of the endoscope and transferred to a PC at 30 frames per second using a USB-ECPT video capture device (Sabrent, Los Angeles, CA, USA).

The image correction software was developed in C++. The sensor data were acquired by a polling method.

### Technical evaluation

To evaluate the performance of the image orientation correction system, a board with a black upper half and a white lower half was placed 30 cm from the PC camera (S101 Web Cam; Kodak, Rochester, NY, USA), and the sensors were attached to the camera. The rotational angle (*θ*_optical_) was automatically calculated from the image by recognizing the black and white boundaries.

While the camera was rotated randomly for 30 seconds, the rotational angles from the accelerometer (*θ*_accel_), gyrosensor (*θ*_gyro_), and Kalman filter (*θ*_Kalman_), the postprocessed rotational angle (*θ*_postproc_), and the optical rotational angle (*θ*_optical_) were recorded. Every measurement was repeated using the sensor’s wired and wireless connection.

### Clinical utility test

**Fig 3:**
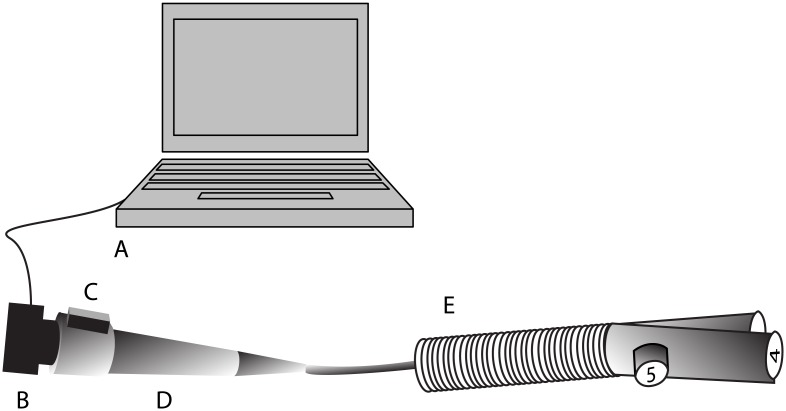
Experimental design of the clinical utility test. Participants reported numbers printed on papers at the left main, right main, and right upper bronchi of the airway model for both exams with and without using the image orientation correction system. The numbers inside the airway model were reset for each test. A, computer; B, charged-coupled device (CCD) camera; C, sensor; D, handle of the endoscope; and E, airway model with numbered papers at the left main, right main, and right upper bronchi.

A prospective, randomized observational study using an airway model was used to test the clinical utility of the image orientation correction system. Because the experiment was performed on the airway model and the participants were enrolled as testers, written informed consent was waived. The model simulated human tracheal rings and the angles of the carina and right upper bronchus. Fifteen residents from the Department of Anesthesiology and Pain Medicine at Seoul National University Hospital, each of whom had performed more than 30 bronchoscopic exams, participated in this study. Before the experiment, 5 minutes were allotted for each participant to learn how to use the equipment and practicing with the device. For the experiment, each participant performed two simulated endoscopic exams using two airway tree models in a random order, one with and one without the image orientation correction system (Fig 3). The order of exams was determined by a computer-generated random number. A wired connection was used between the sensor and the computer during the exam with the image orientation correction system.

During each exam, participants verbally reported the printed numbers in the order of left main, right main, and right upper bronchi of the airway model. The correctness and the total time taken to report the numbers were recorded. The numbers inside the airway model were reset for each exam.

For a statistical power of 0.8 and a significance level of 0.05, the estimated sample size was 12, with the assumption that the mean difference and standard deviation between two measurements was 20 seconds and 25 seconds, respectively. Fifteen participants were needed to compensate for possible dropouts. The times required to finish the exam with and without using the image orientation correction system were compared using the Mann-Whitney *U* test. All statistical analyses were performed using SPSS software (version 21; SPSS, Inc., Chicago, IL, USA). *P* < .05 was considered statistically significant. Data are expressed as median (interquartile).

## Results

### Technical evaluation

The optical rotational angle measured from the captured image during the random rotation of the camera ranged from −83.1° to 88.0° with the wired sensor connection and from −108.0° to 90.9° with the wireless connection. The optical angular velocity of the camera ranged from −184°/s to 182°/s with the wired sensor connection and from −251°/s to 201°/s with the wireless connection. The mean sampling rate of the sensor was 333.4 Hz and 27.7 Hz for the wired and wireless connection, respectively.

**Fig 4:**
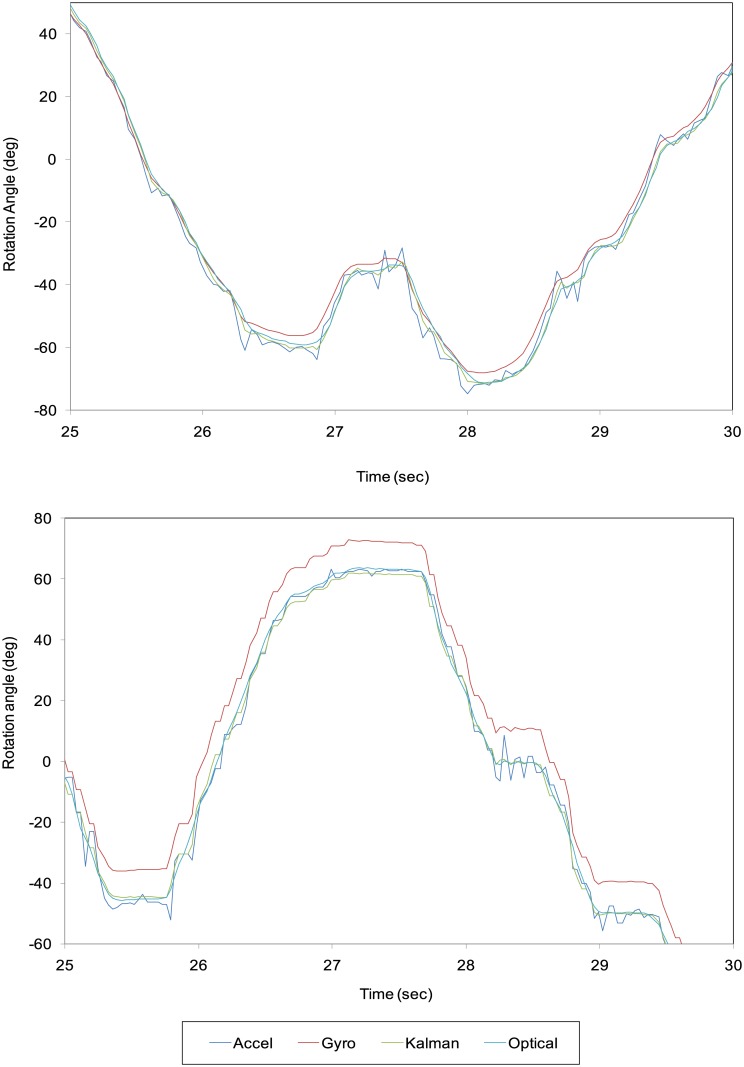
Results of the measured and optical rotational angles from 25 to 30 seconds after beginning the experiments. A, wired connection; B, wireless connection.

Using only the accelerometer output, the maximum difference between *θ*_optical_ and *θ*_accel_ was 12.79° with the wired connection and 15.08° with the wireless connection (Table 1; Fig 4). Using only the gyrosensor output, the maximum difference between *θ*_optical_ and *θ*_gyrol_ was 8.87° with the wired connection and 14.18° with the wireless connection. After the Kalman filter and postprocessing, the maximum difference between *θ*_optical_ and *θ*_postproc_ was 5.00° with the wired connection and 7.48° with the wireless connection.

**Table 1:** Error of rotational angle with different combinations of sensors, connections, and processing. Error was defined as the difference between the measured rotational angle and the optical rotational angle. RMS, root mean square; Kalman, Kalman filtering.

Sensors	Error range (°)	Error RMS (°)
Wired+accelerometer	−10.82 to 12.79	3.55
Wired+gyrosensor	−7.17 to 8.87	3.01
Wired+Kalman	−4.59 to 5.15	2.42
Wired+Kalman+postprocessing	−5.00 to 3.75	1.21
Wireless+accelerometer	−14.92 to 15.08	3.48
Wireless+gyrosensor	−5.07 to 14.19	5.09
Wireless+Kalman	−7.38 to 7.82	2.72
Wireless+Kalman+postprocessing	−7.53 to 7.48	2.70

As shown in Table 1, the minimum root mean square (RMS) error between the calculated and measured optical rotational angles was obtained using the wired connection for both the accelerometer and the gyrosensor with Kalman filtering and postprocessing for the time delay. The wired connection provided better results than the wireless connection for both sensors in most cases.

The time differences between the last available sensor output and image frame measured by Eq (9) were 0.025 and 0.002 seconds for the wired and wireless connection, respectively.

### Clinical utility test

All participants successfully completed the experiments. The time required to finish the bronchoscopic exam decreased significantly using the image orientation correction system (median, 52 seconds; interquartile range, 32–74 seconds) compared with not using the system (median, 76 seconds; interquartile range, 59–128 seconds; *P* = .012).

There was one case when the image orientation correction system was not used in which the number printed at the opposite bronchus was reported.

## Discussion

In the present study, we developed and evaluated an endoscopic image orientation correction system using an accelerometer and gyrosensor. As a result, there was a clear benefit from using the orientation correction system.

**Fig 5:**
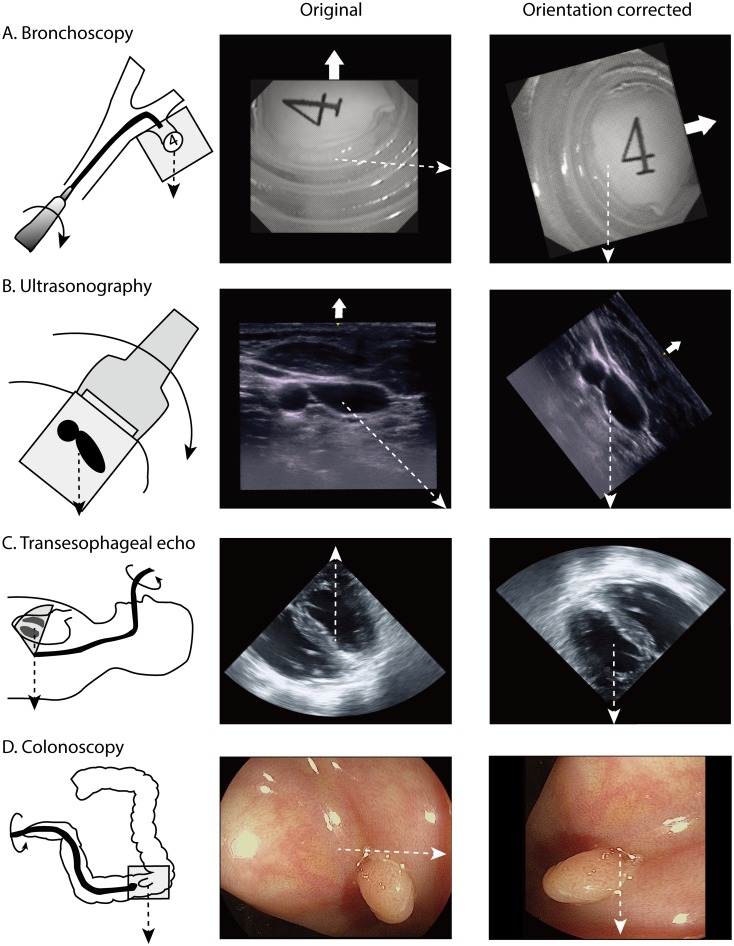
Images before and after orientation correction in various clinical situations. A. With flexible endoscopy, changing the direction requires axial rotation of the endoscope. The gravitational distortion due to the rotation can be corrected by the orientation correction system. The procedure is also easier because the direction of the endoscope tip coincides with the spatial orientation of the endoscopist. This image was captured using the orientation correction system used in this study. B. With ultrasound-guided catheter insertion, the image orientation correction system can help to match the spatial orientation of the image and the operator. This image was captured using the orientation correction system used in this study. C. With conventional transesophageal echocardiography, the direction of gravity is upward. Therefore, air bubbles come down from the top of the image. D. In colonoscopy, the location of the mass can be accurately described even when there is no structural landmark. The upper direction of the raw image is indicated by a thick white arrow and the direction of gravity is indicated by a dashed line.

The direction of a flexible endoscope needs to be changed by rotation, because the tip can only be bent up or down. During ultrasonography, the probe should be positioned obliquely in order to transmit the beam to the target. These changes cause unwanted rotation of the image orientation (Fig 5). By correcting the image orientation relative to the direction of gravity, the image can be interpreted more intuitively. Video clips of the airway model bronchoscopic exam with and without image orientation correction are provided as supplementary files (S1 and S2 Video Files). The orientation corrected image was much easier to interpret because the direction of the image did not change during the exam.

To correct the endoscopic image orientation, some researchers have reported using an accelerometer alone [7, 14]. Although the accuracy of the device in that report was within 1°, this result was achieved only in a stationary state. When using the accelerometer alone in our study, the error was much greater than 1° while the endoscope was rotated. This error decreased significantly when the gyrosensor and accelerometer were used simultaneously. Use of accelerometer and gyrosensor in endoscopic guidance have been reported already [15, 16]. Behrens and colleagues used accelerometer and gyrosensor for endoscope navigating system. In the report, the mean error of the position measurement was between 1 and 4 degrees [15]. Ren, et al. reported that accelerometer and gyrosensor can improve the accuracy of existing electromagnetic tracking system [16]. However, relatively little effort has been made to evaluate the clinical usefulness of this technology.

A novel postprocessing method was also suggested in this study, wherein the time difference between the last available sensor output and image frame multiplied by the angular velocity was subtracted from the Kalman filter output. In the devices used in this study, the time difference between the last available sensor output and image frame was 0.025 and 0.002 seconds with the wired and wireless connection, respectively. Not compensating for this time difference can result in large error, especially with the wired connection. The possibility of postprocessing using the angular velocity is another benefit of using the gyrosensor.

The main advantage of the system developed in this study is that the sensor module can be attached to the handle of the endoscope. While this can cause an error when the tip and handle of a flexible endoscope twist, this is not common in most clinical situations. Also, sensors attached to the tip may increase the tip size and the risk of aspiration when the equipment is damaged. Additionally, attaching the sensor to a conventional endoscope handle is more cost-effective than purchasing new equipment.

This endoscopic image orientation correction system can also help clinicians to objectively analyze the endoscopic images after the procedure. This is particularly useful when the endoscopist and the operator are not the same person (e.g., laparoscopic surgery or transesophageal echocardiography-guided valvuloplasty), when there is no information about the image orientation (e.g., intravascular sonography or epiduroscopy), or when communicating with another specialist for consultation or education.

This study does have some limitations. A camera was used instead of an endoscope during the technical evaluation phase. However, measuring the optical rotational angle from with an actual endoscope may have induced an error due to the lower resolution. Second, an error may have occurred during the technical evaluation if the center of the camera did not align exactly with the center of the target board. Third, an airway model was used instead of an animal or human airway for the clinical utility test. During a bronchoscopic exam of a human patient, the shape of tracheal rings and muscle stripes can help orient the operator; however, the airway model used in this study did not have such detailed structures.

In conclusion, the endoscopic image orientation correction system using both an accelerometer and a gyroscope resulted in greater accuracy than using an accelerometer alone. This system also significantly decreased the time needed to perform a bronchoscopic exam, which would be quite valuable in the clinical setting. Although these results were obtained in a limited situation, this orientation correction system will likely help clinicians interpret and analyze endoscopic images in clinical practice.

## Supporting information

S1 Video File Captured video without using the endoscopic image correction system. As the endoscope rotates, the image also rotates, making it difficult to interpret the direction.
(AVI)

S2 Video File Captured video with the endoscopic image correction system. The orientation of the image is fixed to the direction of gravity while the endoscope is rotating. A wired connection was used for capturing the video.
(AVI)
